# Molecular Mechanisms of Diabetic Retinopathy, General Preventive Strategies, and Novel Therapeutic Targets

**DOI:** 10.1155/2014/801269

**Published:** 2014-07-06

**Authors:** Sher Zaman Safi, Rajes Qvist, Selva Kumar, Kalaivani Batumalaie, Ikram Shah Bin Ismail

**Affiliations:** Department of Medicine, Faculty of Medicine, University of Malaya, 50603 Kuala Lumpur, Malaysia

## Abstract

The growing number of people with diabetes worldwide suggests that diabetic retinopathy (DR) and diabetic macular edema (DME) will continue to be sight threatening factors. The pathogenesis of diabetic retinopathy is a widespread cause of visual impairment in the world and a range of hyperglycemia-linked pathways have been implicated in the initiation and progression of this condition. Despite understanding the polyol pathway flux, activation of protein kinase C (KPC) isoforms, increased hexosamine pathway flux, and increased advanced glycation end-product (AGE) formation, pathogenic mechanisms underlying diabetes induced vision loss are not fully understood. The purpose of this paper is to review molecular mechanisms that regulate cell survival and apoptosis of retinal cells and discuss new and exciting therapeutic targets with comparison to the old and inefficient preventive strategies. This review highlights the recent advancements in understanding hyperglycemia-induced biochemical and molecular alterations, systemic metabolic factors, and aberrant activation of signaling cascades that ultimately lead to activation of a number of transcription factors causing functional and structural damage to retinal cells. It also reviews the established interventions and emerging molecular targets to avert diabetic retinopathy and its associated risk factors.

## 1. Introduction

The number of people with diabetes worldwide was 382 million in 2013 and nearly 592 million people are estimated to be diabetic by 2035 [1]. Diabetes is one of the most common metabolic disorders, characterized by defective secretion of insulin. Immune mediated destruction of pancreatic b-cells leads to insulin deficiency and eventually to type I diabetes, while type II diabetes is characterized by insulin resistance and relative deficiency in insulin signaling [2]. Hyperglycemia is recognized as a major responsible factor for the development of diabetic complications. Diabetes involves many overlapping and interrelated pathways that results in potentially blinding complications like diabetic retinopathy and macular edema [3]. Diabetic retinopathy (DR) is the most widespread microvascular complication of diabetes and a major cause of vision loss worldwide. Globally, there are approximately 93 million people with DR, 17 million with proliferative DR, 21 million with diabetic macular edema, and 28 million with VTDR [4]. A new systematic review of 35 population-based studies has revealed that the prevalence of diabetic retinopathy, proliferative diabetic retinopathy (PDR), and diabetic macular edema (DME) among diabetic patients is 34.6%, 7.0%, and 6.8%, respectively [5, 6]. It is characterized by the increased development of distinct morphological abnormalities in the retinal microvasculature that either remains stable or progresses to diabetic macular edema or proliferative diabetic retinopathy, which are leading causes of severe visual impairment in working-age adults especially in industrialized countries [7]. The severity of diabetic retinopathy ranges from nonproliferative and preproliferative to more severely proliferative diabetic retinopathy, in which the abnormal growth of new vessels occurs [8]. A number of clinical trials on the prevention or treatment of diabetic retinopathy and diabetic macular edema (DME) are in progress (Table 1).

Multiple cellular pathways and potential molecular mechanisms have been proposed to explain diabetes induced complications. In diabetic retinopathy some of the most studied mechanisms are increased polyol pathway flux, increased advanced glycation end-products (AGE) formation, abnormal activation of signaling cascades such as activation of protein kinase C (PKC) pathway, increased oxidative stress, increased hexosamine pathway flux, and peripheral nerve damage. All these pathways in one way or another end in increased oxidative stress, inflammation, and vascular occlusion, causing upregulation of factors such as insulin-like growth factor (IGF), stromal derived factor-1 (SDF-1), vascular endothelial growth factor (VEGF), angiopoietins (Ang-2), tumor necrosis factor (TNF), and basic fibroblast growth factor-2 (bFGF) that eventually contribute to the pathogenesis of diabetic retinopathy [10, 11].

A number of candidate genes have been identified which are directly or indirectly involved in diabetic retinopathy. Aldose reductase (ALR2), endothelial nitric oxide synthase (eNOS), vascular endothelial growth factor (VEGF), receptor for advanced glycation end products (RAGE), paraoxonase1 (PON1), angiotensin converting, and plasminogen activator inhibitor1 (PAI) are some of the genes that are shown to be associated with diabetic retinopathy. Several polymorphisms at the regulatory regions of these genes have been characterized and evaluated as risk alleles for the susceptibility or progression of diabetic retinopathy in different populations of the world [12, 13]. Hypertension, hyperglycemia, and diabetes duration are the established risk factors of diabetic retinopathy. The diabetes control and complications trial (DCCT) conducted a study in 1993 to see whether intensive or conventional method is more efficient. According to their report the intensive treatment and improved glucose control delayed the onset of retinopathy and slowed down its progression in comparison to conventional method of treatment [14]. Intensive glucose and blood pressure control can lessen the progression of diabetic retinopathy but long-term management of these risk factors could be difficult to manage. Laser photocoagulation and Focal/grid photocoagulation have been shown to be effective in treating and reducing further vision loss [15]; however, these procedures are associated with potential complications, affecting visual field, color vision, and contrast sensitivity [16]. To avoid all these complications, new drugs and therapeutic targets must be identified which can disrupt the chain of events that lead to vision loss and weakening of the retina. In this paper we have tried to review molecular mechanisms regulating cell survival and apoptosis of retinal cells and discuss new and exciting therapeutic targets with comparison to the old and inefficient preventive strategies.

## 2. Molecular and Biochemical Mechanisms of Diabetic Retinopathy and Its Pathogenesis

**Figure 1:**
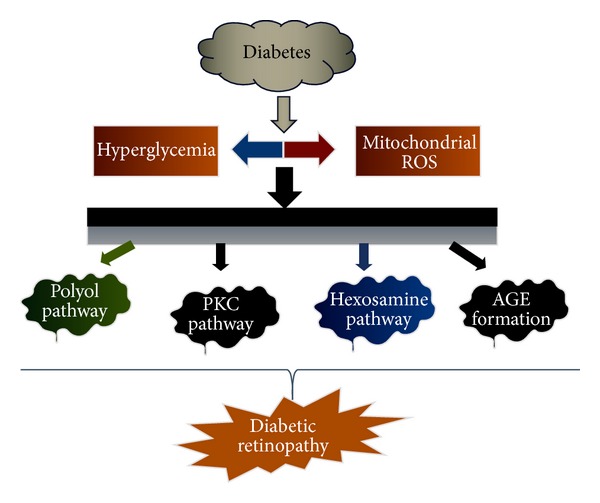
The four major mechanisms involved in DR are increased polyol pathway flux, increased AGE formation, activation of PKC, and polyol pathways.

**Figure 2:**
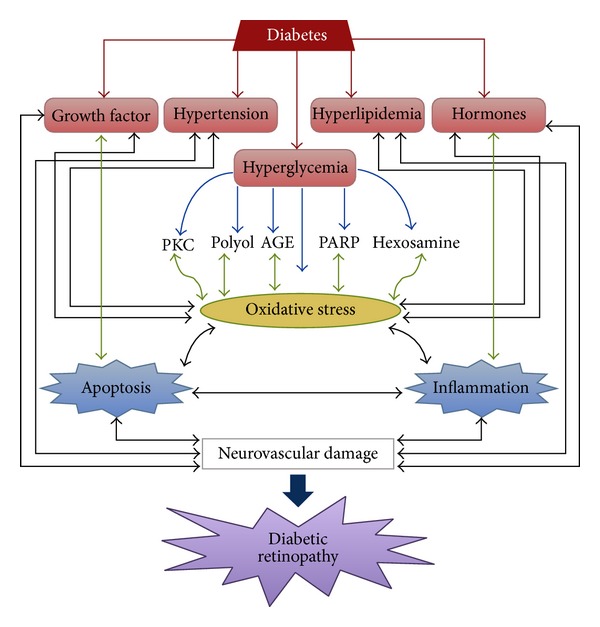
Hyperglycemia-induced biochemical alterations precipitated by mitochondria-driven oxidative stress leading to diabetic complications including apoptosis, inflammation, and ultimately diabetic retinopathy.

A range of studies have described the biochemical mechanisms in the development and progression of diabetic retinopathy; however, no mechanism can be regarded as established. All forms of diabetes are characterized by hyperglycemia, insulin resistance, relative or absolute lack of insulin action, and the development of diabetes specific pathology in the retina [17]. Diabetic retinopathy has been one of the major factors of vision impairment in the world. The basic hallmarks of this disease include loss of pericytes, basement membrane thickening, microaneurysms, neovascularization, and blood retinal barrier breakdown [18]. Molecular and biochemical mechanisms that have been implicated in diabetic retinopathy are increased flux of glucose through the polyol and hexosamine pathways, activation of protein kinase C, and increased advanced glycation end product formation [19] (Figures 1 and 2).

### 2.1. Increased Polyol Pathway Flux

**Figure 3:**
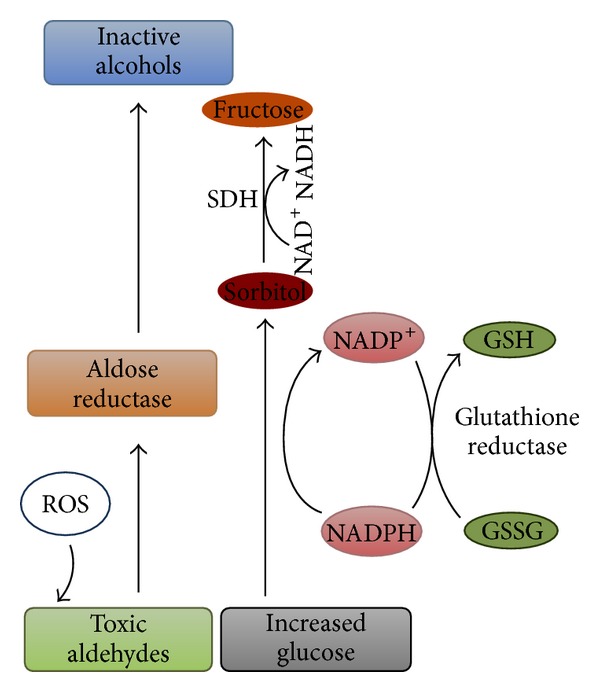
Aldose reductase and the polyol pathway. Aldose reductase reduces aldehydes generated by reactive oxygen species (ROS) to inactive alcohols, and glucose to sorbitol, using NADPH as a cofactor. Glutathione (GSH), glutathione disulfide (GSSG), and sorbitol dehydrogenase (SDH).

Detrimental effects of hyperglycaemia-induced increase in polyol pathway flux could be explained by a number of proposed mechanisms including decreased (Na^+^&K^+^) ATPase activity, sorbitol-induced osmotic stress, decrease in cytosolic NADPH, and increase in cytosolic NADH/NAD^+^. The polyol pathway is a two-step metabolic pathway in which glucose is reduced to sorbitol, which is then converted to fructose (Figure 3). Several biochemical and molecular studies implicate the polyol pathway as a reasonable and significant contributor to diabetic retinopathy and other complications of diabetes. Retinal endothelial cells of both rat and human showed aldose reductase immunoreactivity and human retinas exposed to high glucose in organ culture increased the production of sorbitol by a degree comparable to that observed in the rat. Such excess aldose reductase activity can be a mechanism for human diabetic retinopathy [20].

The polyol pathway of glucose metabolism becomes active when intracellular glucose levels are elevated. Aldose reductase (AR), the first and rate-limiting enzyme in the pathway, reduces glucose to sorbitol using NADPH as a cofactor; sorbitol is then metabolized to fructose by sorbitol dehydrogenase, which uses NAD^+^ as a cofactor [21, 22]. Under euglycemic conditions, the higher affinity of hexokinase for the glucose substrate ensures the formation of sorbitol at a low level. However, in hyperglycemic conditions, there is a substantial increase in intracellular sorbitol levels. Aldose reductase has a high capacity and a low affinity for glucose, but sorbitol dehydrogenase (SDH) has a high affinity and a low capacity for sorbitol. Thus, glucose flux mediated by Aldose reductase is very low in this pathway except during hyperglycemia, and sorbitol oxidation is relatively independent of the sorbitol concentration within the physiological range [23, 24]. In diabetes, the sorbitol pathway increases in activity in tissues like retina, kidney, peripheral nerves, and blood vessels where insulin is not required for cellular glucose uptake. Sorbitol does not easily diffuse through cell membranes; as a result, it accumulates and causes osmotic damage [25].

The polyol pathway is by all criteria an extremely attractive target for the treatment of diabetic retinopathy; however, we cannot exclude the possibility of other mechanisms of polyol pathway-induced damage only in few particular types of retinal cells. For example, osmotic stress seemingly cannot be invoked from data in the whole retina because retinal levels of sorbitol increase in diabetic rats only 3–8-fold above control [26, 27]. Although animal data persuasively shows that aldose reductase has an early role in the pathogenesis of diabetic retinopathy, studies of inhibition of the polyol pathway in vivo have yielded inconsistent results. The long-term sorbinil trial [28] also indicated that sorbinil did not prevent the worsening of the disease except for a slower progression rate. The failure in clinical trials of therapeutic agents based on these putative pathogenic mechanisms may not rule out the mechanisms as important to the development or progression of diabetic retinopathy.

### 2.2. Accumulation of AGEs

It has become established that chronic exposure of the retina to hyperglycemia gives rise to accumulation of advanced glycation end products (Figure 2) that play an important role in retinopathy [29]. Advanced glycation end products (AGEs) are proteins or lipids that become nonenzymatically glycated and oxidized after exposure to aldose sugars [30]. Some of the best chemically characterized AGEs in human are carboxyethyllysine (CEL), carboxymethyllysine (CML), and pentosidine, which are shown to play a crucial role in the formation and accumulation of AGE in hyperglycemia. CML and other AGEs have been localized to retinal blood vessels of diabetes patients and were found to correlate with the degree of retinopathy suggesting the pathophysiological role of AGEs in diabetes [31]. Early glycation and oxidation processes result in the formation of Schiff bases and Amadori products. Further glycation of proteins and lipids causes molecular rearrangements that lead to the generation of AGEs [32]. These AGEs contribute to a variety of microvascular and macrovascular complications through the formation of cross-links between molecules in the basement membrane of the extracellular matrix and receptor for advanced glycation end products (RAGE) [33]. After cellular attachment, AGEs have been shown to increase procoagulant activity, vascular permeability, adhesion molecule expression, and monocyte influx actions that may contribute to vascular injury [34].

A number of studies have reported that oxidative stress is increased in diabetic patients and that it plays an important role in the pathogenesis of diabetic complications, including diabetic retinopathy [35]. It has been reported that overproduction of mitochondrial superoxide dismutase (SOD) [36] and inhibition of superoxide with antioxidants [37, 38] can protect against capillary degeneration during diabetic retinopathy in experimental diabetes, although how this influences AGE accumulation in the retina has not been studied. It is also possible that some chelators may shift the redox potential of iron or copper, affecting their catalytic activity in a way that could exacerbate oxidative stress and diabetes complications. The involvement of inflammatory processes in the initiation of neurovascular lesions during diabetic retinopathy has received recent attention. Global mRNA expression profiling has highlighted altered expression of proinflammatory cytokines and interrelated pathways, not only in the retinal vessels, but also in the neuroglia [39]. A study in which they investigated the correlation between age, metabolism, and AGEs found that older adults had higher serum concentrations than younger adults; however, the authors also reported that higher AGE concentrations were directly related to dietary intake across all age groups [35].

Increasing evidence suggests that AGE receptor binding can initiate important signaling pathways involving tyrosine phosphorylation of Janus kinase (JAK)/signal transducers and activators of transcription (STAT) [36], recruitment of phosphatidylinositol 3′ kinase to Ras [37], activation of protein kinase C [38], and oxidative stress through NFkB and AP-1 transcription [39]. AGEs interact with cells through several routes. AGE-modified serum proteins, such as CML, may interact with vascular endothelium via RAGE, which can activate nuclear factor kappa B (NF-*κ*B), leading to enhanced expression of adhesion molecules and secretion of cytokines such as tumor necrosis factor alpha (TNF-alpha) and VEGF. Similarly sedentary cells like endothelium encounter AGEs such as pentosidine-derived cross-links within basement membrane proteins where they may disrupt integrin signaling. These reactions, together with intra- and intermolecular cross-link formation, are able to modify structure and function of target molecules in such a way that they do not respond anymore to biological signals [40, 41]. AGEs also inhibit prostacyclin production and stimulate plasminogen activator inhibitor-1 (PAI-1) through an interaction with RAGE [42]. The molecular mechanisms of VEGF overexpression induced by AGEs are not fully understood; however, recent investigations have shown that the AGE-RAGE interaction might increase VEGF gene transcription by NADPH oxidase-mediated ROS generation and the subsequent nuclear factor-*κ*B (NF-*κ*B) activation via Ras-mitogen activated protein kinase (MAPK) pathway [43, 44]. In another recent study it has been shown that knocking down of integrin-linked kinase (ILK) gene expression with siRNA inhibited the elevation of VEGF and intercellular adhesion molecule 1 (ICAM-1). These results suggest that ILK has been involved in the response of cells to high glucose and may therefore play a role in the pathogenesis of diabetic retinopathy [45].

In a study when AGE-modified albumin was administered to nondiabetic rats for 4 weeks, it caused glomerular hypertrophy and increased extracellular matrix production in association with activation of the genes for collagen, laminin, and TGFb [46]. Other observations in diabetic animals are compatible with a pathogenetic role for AGEs in microvascular disease. Similarly AGEs alone are given to achieve plasma concentrations equivalent to those seen in diabetic animals [47]. After 5 months, the renal AGE content in AGE-treated rats was 50% above that in controls, while the plasma concentration was 2.8 times greater than that of controls. It is well established that AGEs are involved in the pathogenesis of diabetic complications. However, more studies are needed to elucidate the exact role of AGE in this area.

### 2.3. Increased Flux through the Hexosamine Pathway

Hexosamine content has been found to be increased in retinal tissues of humans and rats with diabetes [48]. Recent in vitro and in vivo studies have revealed that the increased flux of glucose via the hexosamine pathway has been implicated in insulin resistance, diabetic vascular complications (Figure 2), and stimulation of the synthesis of growth factors [49, 50]. In particular, it was demonstrated that hyperglycemia-induced production of transforming growth factor-b (TGF-b1), a prosclerotic cytokine, was causally involved in the development of diabetic nephropathy. In the hexosamine pathway, fructose-6-phosphate is converted to N-acetylglucosamine-6-phosphate by glutamine fructose-6-phosphate amidotransferase (GFAT). N-Acetylglucosamine-6-phosphate is then converted to N-acetylglucosamine-1,   6-phosphate, and UDP-GlcNAc. UDP-GlcNAc is a substrate for O-linked glycosylation, which is catalyzed by O-GlcNAc transferase. UDP-GlcNAc, the major product, is the unique donor for the O-linkage of a single N-acetylglucosamine molecule (O-GlcNAc) to many cytoplasmic and nuclear proteins [51, 52]. Glucose is rapidly phosphorylated to glucose-6-phosphate after entering the cell which can then oxidize via glycolysis or the pentose phosphate shunt or stored as glycogen. Before the pathway proceeds, G6P is isomerized to fructose-6-phosphate (F6P) during glycolysis. Fructose-6-phosphate-amidotransferase (GFAT) catalyzes the formation of glucosamine-6-phosphate with glutamine as an amine donor and F6P as an acceptor substrate in the first and rate-limiting step of the pathway [53, 54].

Inhibition of the rate-limiting enzyme in the conversion of glucose to GFAT blocks hyperglycaemia-induced increases in the transcription of TGF-b1 [55] and plasminogen activator inhibitor-1 (PAI-1) [56]. Chen et al. observed that binding sites for the transcription factor Sp1 regulate hyperglycaemia-induced activation of the PAI-1 promoter in vascular smooth muscle cells which suggests that covalent modification of Sp1 by N-acetylglucosamine (GlcNAc) might explain the link between activation of the hexosamine pathway and hyperglycaemia-induced changes in transcription of the gene for PAI-1 [57]. Thus, activation of the hexosamine pathway by hyperglycaemia results in many changes in both gene and protein levels, which together contribute to the pathogenesis of diabetic retinopathy. Another study [58] suggests that the increased glucose flux by the hexosamine pathway may direct retinal neurons to undergo apoptosis in a bimodal fashion, that is, via induction of apoptosis possibly by altered glycosylation of proteins and via perturbation of the neuroprotective effect of insulin mediated by Akt. This report emphasizes that hexosamine pathway may be involved in retinal neurodegeneration in diabetes. Clinically, the increased GFAT activity has been well interrelated with HbA1c levels in diabetic patients [59]. Distinct and high expression of GFAT was also demonstrated in diabetic nephropathy and other complications [52]. Despite a number of studies and data documentation saying that most of the tissues express GFAT, in eye specific tissues the data on GFAT is still lacking [51].

### 2.4. PKC Pathway

Increased vascular permeability and excessive neovascularization are the hallmarks of endothelial dysfunction, which can lead to diabetic macular edema and proliferative diabetic retinopathy. Many of the microvascular alterations in the eyes of patients with diabetes are thought to arise from hyperglycemia-induced activation of protein kinase C (PKC). PKC consists of a family of multifunctional serine/threonine kinases, which are involved in the control of other proteins. So far, at least 12 PKC isoforms have been identified and can be subdivided into three groups: classical, novel, and atypical. The activities of the classical isoforms (PKC-*α*, -*β*1/2, and PKC-*δ*) are greatly enhanced by DAG and have been linked to vascular dysfunctions and pathogenesis of DR [51]. Being important signaling transducers, PKCs are activated when second messengers bind to their regulatory domain, usually at the plasma membrane [60]. Similar vascular pathological conditions are observed in diabetic animals and those with diet-induced hypergalactosemia. Both diabetes and hypergalactosemia are believed to cause vascular dysfunction via a common biochemical mechanism. Out of twelve PKC isoforms so far identified, nine are activated by the lipid second messenger DAG, and this implies that altered DAG-PKC pathway may have an important role in diabetic complications [61]. In cultured microvascular cells and in the retina and renal glomeruli of diabetic animals, intracellular hyperglycaemia increases the amount of DAG and increased de novo synthesis of DAG results in activation of PKC [62]. PKC activation can lead to mitogen-activated protein kinase (MAPK) activation and phosphorylation of several important transcription factors that increase gene expressions of various stress related genes like c-Jun kinases and heat shock proteins [63]. PKC-b has been shown to have a role in the form of a signaling component for VEGF and a regulator of endothelial cell permeability [64]. Furthermore, PKC activation contributes to the overexpression of plasminogen activator-1 (PAI-1), the activation of NADPH oxidase, and the activation of NFkB in many vascular cells including endothelial cells, smooth muscle cells, pericytes, mesangial cells, and others [65]. Thus, PKC activation involving several isoforms is likely to be responsible for some of the pathologies in diabetic retinopathy.

### 2.5. Relationship of These Pathways

All these mechanisms and events act in conjunction in several ways. Hyperglycemia activates polyol pathway, where a part of excess glucose is metabolized to sorbitol which is then changed to fructose. Aggregated sorbitol within retina may cause osmotic stress and also the byproducts of polyol pathway, fructose-3-phosphtae, and 3-deoxyglucosone are powerful glycosylating agents that result in the formation of AGEs [66]. Similarly AGE formation leads to activation of PKC pathway and poly (ADP-ribose) polymerase that may lead to initiation of inflammation and growth factor imbalances [67]. An increased flux through hexosamine pathway is associated with TGF-*β* expression, PKC activation, and ECM production, all of which are linked with the pathogenesis of diabetic retinopathy [68].

## 3. General Preventive Strategies 

General strategies for the prevention of diabetic retinopathy should be aimed at identifying risk factors in the patient and counseling for the same. Patients should be encouraged to keep a close eye on their blood pressure, duration of the disease, hyperlipidemia, metabolic control, hypertension, and family history. For reducing and restoring the vision loss, scatter (panretinal) laser photocoagulation or vitrectomy surgery can also be employed.

### 3.1. Primary Prevention

**Figure 4:**
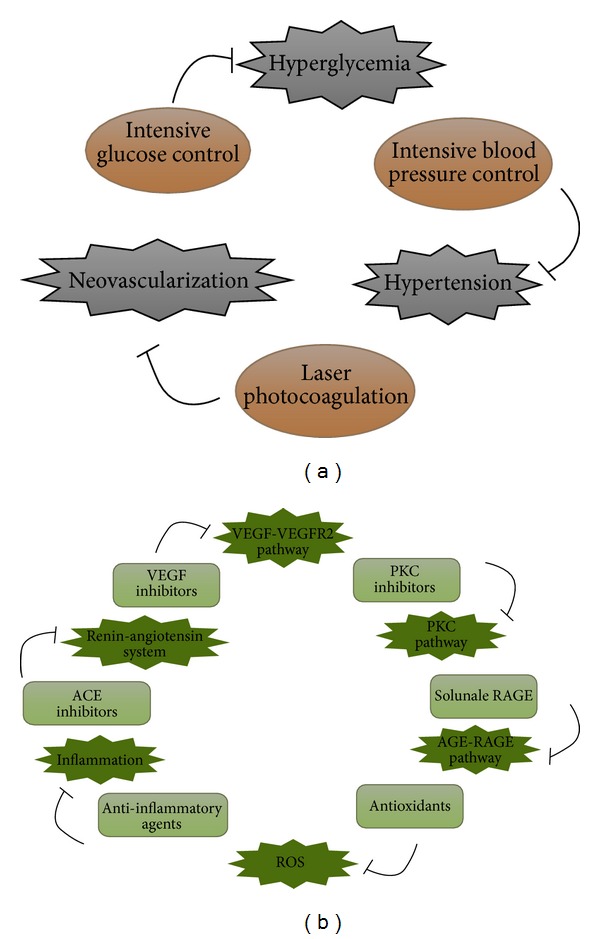
(a) The established preventive measures including general, primary, and secondary preventive strategies. (b) Novel and emerging therapeutic targets including PKC inhibitors, VEGF inhibitors, ACE inhibitors, and drugs as antioxidants.

The initial approach in diabetes management is lifestyle modifications. For instance, modifications before administrating medication, a healthy dietary pattern and physical activity program are the mainstay of diabetes treatment. In type diabetes, healthy eating habits, along with healthy weight, normal lipids level, and good control of blood glucose, are the basic goals to be considered. Regular exercise, healthy food choices, and weight loss are the basic lifestyle modifications in type 2 diabetes. The diabetes control and complications trial (DCCT) from 1983 to 1993 [14] established that intensive control of blood glucose levels for type 1 diabetes considerably reduces the risk of onset and progression of retinopathy and the need for laser surgery. It was demonstrated by reduction of glycated hemoglobin readings. Intensive control also had a beneficial effect in reducing the risk of kidney disease, neuropathy, and to a lesser degree, large vessel disease. Although intensive glucose and blood pressure control can reduce diabetic retinopathy (DR) progression, the long-term management of these risk factors over decades of diabetes duration can be difficult to maintain. Evidence from randomized controlled trials (RCTs) indicates that tight control of blood pressure is a major modifiable factor for the incidence and progression of DR. According to several observational studies, dyslipidemia also increases the risk of DR, particularly DME [69, 70] (Figure 4(a)).

### 3.2. Secondary Prevention

Current treatment modalities, precise clinical algorithms for diagnosis, management, follow-up, and understanding of diabetic retinopathy have largely reduced the risk of vision loss from both diabetic retinopathy and diabetic macular edema. Laser photocoagulation and vitrectomy have improved the quality of life for patients with diabetic retinopathy and prevented the visual loss [71]. In the 1970s and 1980s, randomized controlled clinical trials (RCTs) in diabetic retinopathy began to evaluate the effects of laser photocoagulation [72, 73]. The Early Treatment Diabetic Retinopathy Study (ETDRS) (1979–1990) also elucidated the natural history of diabetic retinopathy and provided new insights into the optimization of scatter laser photocoagulation for diabetic retinopathy [74]. These studies have helped in defining and fixing the natural history of diabetic retinopathy but at the same time highlighted the wide intersubject and intrasubject variability in retinal appearances and clinical progression. The Diabetic Retinopathy Study (DRS) (1971–1975) established the benefits of scatter (panretinal) laser photocoagulation for reducing the risk of vision loss from proliferative diabetic retinopathy [75]. From 1977 to 1987, the Diabetic Retinopathy Vitrectomy Study (DRVS) demonstrated the value of vitrectomy surgery for restoring useful and optimal timing for vitrectomy and for eyes with nonresolving vitreous hemorrhage or traction retinal detachment [76]. However, laser photocoagulation and vitrectomy are implicated only when DR has progressed to a measurably advanced stage in which some visual loss has already occurred. Because of these limitations of current management strategies, new pharmacological therapies are being developed, targeting the fundamental pathogenic mechanisms that initiate or sustain the progression of DR (Figure 4(a)).

## 4. Emerging Therapeutic Therapies 

Early detection of retinal abnormalities is essential in preventing diabetic retinopathy (DR) and consequently loss of vision. Options for treating diabetic retinopathy are limited and display poor efficacy. So far the recommended treatment for DR is laser photocoagulation; however, this procedure also destroys the neural tissues [77]. Therefore, we need to establish clinical, biochemical, and molecular research methodologies to develop novel therapeutic strategies for diabetic retinopathy. Newer therapeutic mechanisms are under investigation including PKC pathway inhibitors, VEGF antagonists, AGE pathway inhibitors, and hormone antagonists. These new therapeutic options should result in better management and outcome of diabetic retinopathy (Figure 4(b)).

### 4.1. AGE Inhibitors and Prevention of Retinopathy

In hyperglycemic conditions, carbohydrates interact with protein side chains in a nonenzymatic way to form Amadori products, which consequently form AGEs. Excessive formation of AGEs is considering a biochemical link between diabetes and the development of diabetic retinopathy [78]. The chronic interaction of these products with AGE-specific receptors may perpetuate a proatherosclerotic state and proinflammatory signaling process in vascular tissues. Interaction of AGE-AGE-specific receptors has also been linked with the activation of nuclear factor-*κ*B and oxidative stress, which leads to hyperexpression of lymphocyte adhesion molecules, proinflammatory cytokines, procoagulant factors, and vasoactive mediators [30]. Current treatments focus on preventing the formation of advanced glycosylation end products, breaking established crosslinks and reducing their receptor-ligand interactions.

A variety of different compounds that inhibit advanced glycation end products (AGEs) have been under investigation. Several approaches seeking to reduce AGE interactions, either by inhibiting AGE formation, blocking AGE action, or breaking preexisting AGE cross-links, have been explored. Recently Park et al. [30, 79, 82] have identified a novel inhibitor of the Wnt pathway that is a pigment epithelium-derived factor (PEDF), a multifunctional serine proteinase inhibitor. They have reported that overexpression of PEDF in transgenic mice as well as administration of PEDF protein attenuated Wnt signaling induced by retinal ischemia. In another recent study, Sheikpranbabu et al. investigated the PEDF and reported that PEDF abrogates AGE-induced oxidative stress and apoptosis in retinal pericytes by suppressing nicotinamide adenine dinucleotide phosphate- (NADPH-) oxidase mediated ROS generation and subsequently VEGF expression [83]. The work by Yamagishi and his group has shown that injection of AGEs to normal rats increases RAGE and ICAM-1 expression that induced retinal leukostasis and hyperpermeability; however, the process was blocked by simultaneous treatment with PEDF that completely inhibited superoxide generation and NFkB activation in AGE-exposed endothelial cells [81]. Combination of these studies suggests that PEDF can be an effective therapeutic agent for the treatment of diabetic retinopathy, by abrogating Wnt signaling and AGE-induced oxidative stress in retinal pericytes.

A pharmacologic strategy for AGE-inhibition commenced with the small nucleophilic hydrazine compound called aminoguanidine. This drug is a potent inhibitor of AGE-mediated cross-linking and has been shown to prevent diabetic vascular complications, including diabetic retinopathy, in experimental animals [82]. Blockade of the AGE/RAGE interaction by soluble RAGE has been shown to suppress nephropathy in diabetic animals [83]. sRAGE can prevent Muller cell dysfunction [84] during diabetes and retinal capillary leukostasis in AGE-infused normal mice. The clinical potential for reducing RAGE signaling in the diabetic retina is further underscored by development of new RAGE-regulating agents. One such agent is TTP488, which is an orally delivered small molecule, for which phase II studies have been completed [85].

Diabetes drugs, such as thiazolidinediones and nifedipine or (calcium channel blockers), have been investigated to reduce the expression of RAGE in endothelial cells and could serve to limit the proinflammatory effects of AGEs [86, 87]. Consequently the downregulation of RAGE and associated decrease in oxidative stress can act as PPAR*γ* selective regulators, which suggests some cross-talk between the AGE-RAGE axis and PPAR*γ* modulation. Therefore, a composite interaction between suppressed antioxidant status, enhanced levels of AGEs, and upregulation of the RAGE axis may together play a key role in the progression of diabetic retinopathy [88]. In diabetic animals, AGE inhibitors such as aminoguanidine can attenuate the formation of retinal microvascular lesion; however, recent studies indicate that this drug can significantly alleviate diabetic retinopathic lesions without affecting the formation of the AGE pentosidine in retinal basement membrane collagen [89]. This effect might be linked with aminoguanidine's antioxidative property rather than AGE inhibitory properties [90] as oxidative imbalances in diabetic retinopathy have also been extensively reported [91, 92]. Interestingly, some studies have reported no significant change of retinal vascular lesions after treatment with antioxidants [93]. Therefore, the exact role played by oxidative stress with advanced glycation reactions needs to be examined more intimately.

### 4.2. PKC Inhibitors as Potential Therapeutics

PKC isoform and selective inhibitors are likely new potential therapeutics, which can delay the onset or stop the progression of diabetic vascular diseases. First and second generation PKC inhibitors, for example, isoquinolinesulphonamides and staurosporine, were not even specific for PKC, but as the biochemical and functional profiles of individual PKC isoforms developed there has been renewed interest in the therapeutic opportunities for isoform selective blockade of PKC activation [94]. In 1996, the chemical characterization and in vivo pharmacological profile of orally active and highly selective PKC-*β* inhibitor, ruboxistaurin mesylate (LY333531), were reported in science [95]. The highly selective activation of PKC*β* and its inhibition by ruboxistaurin mesylate have been studied extensively [96] and demonstrated through clinical studies in the prevention of vision loss [97]. In 2009, Geraldes et al. demonstrated that hyperglycemia persistently activated PKC*δ* and p38*α* MAPK to increase the expression of a novel target, SHP-1, leading to PDGF receptor-*β* dephosphorylation and actions, and increased pericyte apoptosis, independent of NF-*κ*B [98]. These findings can be further demonstrated in patients with disease progression, as inhibition of this pathway attenuates the blood retinal barrier breakdown, which is the basis of diabetic retinopathy pathophysiology. Vitamin E is another type of PKC inhibitor: it can inhibit PKC activity probably by decreasing DAG contents via the activation of DAG kinase [99]. High dose vitamin E supplementation has been reported to normalize retinal blood flow in patients with type 1 diabetes [100].

VEGF is a potent proangiogenic factor in a very wide range of pathological conditions. Activation of PKC*β* seems to be an essential step in VEGF mediated endothelial cell migration and replication. Inhibition by ruboxistaurin reduces the mitogenic response to VEGF, in contrast with PKC*α* inhibition [101]. Aiello et al. focused on vascular effects of PKC, while studies in rats confirmed a reduction of VEGF-induced permeability by ruboxistaurin [102]. A specific mechanism for reduction in vascular permeability is suggested by Harhaj et al. [103]. It demonstrated that VEGF-induced tight junction protein phosphorylation, tight junction disassembly, and endothelial cell permeability are mediated by PKC. In a study it was concluded that puerarin exerts significant protective effects against DR in rats, likely regulating angiogenesis factors expressions, and thus may be an effective and promising medicine for treatment of DR [104].

In the presence of diacylglycerol, both novel and conventional PKC isoforms translocate to the membranes of the cells to start crucial biological events. Being an essential enzyme, inhibition of all PKC isoforms with general approaches will originate severe consequences that can put the survival of animals in danger. Therefore, proper selectivity for inhibition is very crucial in the development of a clinically constructive therapeutic PKC inhibitor [105]. An earlier study, using a nonselective PKC inhibiter (PKC412), reported severe toxicity that excludes its clinical applications in diabetic patients [106]. However, such toxic effects are not surprising because PKC activation is indispensable for a range of functions in the heart and kidneys. Therefore, success with PKC*β* inhibitor therapy for established and relatively severe diabetic retinopathy would also raise questions of its adverse effects. Tuttle et al. have also demonstrated in their study that PKC isoform selective inhibiter can be used for chronic clinical treatment with nominal side effects [107]. Ruboxistaurin is an orally active *β*-specific PKC inhibitor and appears to be well tolerated in large phase II and phase III clinical trials of intermediate duration. Despite the encouraging results from animal models, the therapeutic approaches of such drugs for the treatment of diabetic retinopathy raise both hopes and challenges. In the next few years, we are cautiously optimistic, but clearly, more large studies are needed to establish its efficacy for treatment of diabetic retinopathy and other vascular complications in diabetic patients.

### 4.3. Inhibition of Increased Polyol Pathway

The polyol pathway is comprised of two enzymes. Aldose reductase (AR) reduces glucose to sorbitol with the aid of its cofactor NADPH, and sorbitol dehydrogenase (SDH), with its cofactor NAD^+^, converts sorbitol to fructose. In animal models, treatment with AR inhibitors (ARI) was shown to be effective in preventing the development of various diabetic complications, including diabetic retinopathy [108]. Although the exact mechanism is unknown, AR appears to be the possible link between increased polyol pathway activity and the development of some diabetic complications. Therefore, based on the polyol pathway, preventive and therapeutic approaches are used to develop potent inhibiters for diabetic complications [109]. Only new drugs that inhibit aldose reductase with higher efficacy and safety than older drugs will make possible to learn if the resilience of the polyol pathway means that it has a role in human diabetic retinopathy that should not have gone undiscovered. The rate-limiting enzyme of the pathway, aldose reductase, acts on the glucose molecule at the most upstream possible site in the cascade.

Studies on animal models suggest that AR inhibitor, fidarestat, is active in the treatment of diabetic retinopathy. The use of fidarestat, an inhibitor of aldose reductase neutralizes diabetes-associated retinal oxidative stress and poly (ADP-ribose) polymerase formation [110]. This shows an important role for aldose reductase in diabetes and rationale for the development of aldose reductase inhibitors for counteraction of polyol pathway [111]. Similar results were obtained in the rat model with retinal ischemia-reperfusion injury. Fidarestat treatment caused increased cell death and elevated AR expression, coupled with the prevention or alleviation of sorbitol pathway intermediate accumulation [112]. Also in the streptozotocin-diabetic rats, fidarestat treatment significantly decreased concentrations of sorbitol and fructose in the rat retinas. The expression of ICAM-1 mRNA and leukocyte accumulation in the retinas were significantly reduced. Immunohistochemical study also revealed the suppressive effect of fidarestat on the expression of ICAM-1 [113].

In 2009 Drel et al. demonstrated an increase in PARP activity in streptozotocin-induced diabetic rats and PARP inhibitors reduced retinal oxidative-nitrosative stress, glial activation, and cell death in palmitate exposed pericytes and endothelial cells [114]. A double-blind study in patients with diabetic neuropathy by Sima et al. [115] gave exciting evidence of the efficacy of sorbinil, an aldose reductase inhibitor, against morphological signs of degeneration accompanied by a decrease in the nerve sorbitol level and an increase in the nerve conduction velocity. A similar observation was reported by Greene et al. [116] using another aldose reductase inhibitor, FK-366. In addition, the study using Zucker diabetic fatty rats, an animal model of type 2 diabetes, showed that the administration of a combination of four plant extracts inhibited the development of diabetic cataract through the inhibition of AR activity and protein expression in diabetic lenses [117]. However, the retinal microangiopathy developed by dogs fed a 30% galactose diet was just delayed or not prevented at all [118] by the AR inhibitor sorbinil. One group of researchers revealed that sorbinil was metabolized more abruptly in dogs as compared to rats, yielding unpredictably a shorter plasma half-life [119]. Results of the sorbinil retinopathy trial indicated that sorbinil had no clinically important effect on the course of human diabetic retinopathy [28]. Such negative results dampened the enthusiasm in pursuing the polyol pathway as a major player and target in diabetic retinopathy; however, the positive effect of aldose reductase inhibition on diabetic neuropathy with zenarestat [120] provides vested hopes in the use of these compounds in diabetic retinopathy which needs to be tested and validated by future studies.

### 4.4. Inhibition of ROS, Antioxidants, and Hexosamine Pathway as Emerging Therapeutics

Glutamine, fructose-6-phosphate amidotransferase, is the rate-limiting step of the hexosamine biosynthesis pathway, which activates as an alternative pathway to glycolysis for the utilization of hyperglycemia-induced overproduction of fructose-6-phosphate, resulting in excess of N-acetylglucosamine and irregular alteration of gene expression of plasminogen activator inhibitor-1 and TGF-*β*. This overexpression causes a spectrum of adverse metabolic diabetic derangements and endothelial cell and retinal neuron apoptosis [56, 121, 122]. The use of appropriate compounds has been described that can potentially alleviate the metabolic and functional abnormalities in diabetic retinopathy. WAS-406 (2-acetamido-1,3,6-tri-O-acetyl-2,4-dideoxy-*α*-D-xylo-hexopyranose) and Azaserine reduce cardiovascular effects caused by hyperglycemia as antioxidants rather than by inhibiting only the hexosamine pathway [123, 124]. Rhein, an anthraquinone compound isolated from rhubarb, decreases hexosamine pathway and is helpful in treatment of experimental diabetic nephropathy [125]. Benfotiamine, which converts fructose-6 phosphate into pentose-5 phosphates, is another compound that reduces flux through the hexosamine pathway [126]. The ability of benfotiamine, a lipid soluble thiamine, to inhibit simultaneously the hexosamine pathway along with AGE formation and PKC pathways might be clinically useful in preventing the development and progression of diabetic pathogenesis arising due to hyperglycemia-induced vascular damage. In a study, Hammes et al. have shown that benfotiamine can also inhibit hyperglycemia-associated NF-kappaB activation by activating the pentose phosphate pathway enzyme transketolase, which converts glyceraldehyde-3-phosphate and fructose-6-phosphate into pentose-5-phosphates and other sugars [126].

There are several lines of evidence to suggest that antioxidant defenses may be lower in diabetes. These include reports of reduced plasma/serum total antioxidant status or free radical scavenging activity and increased plasma oxidisability in type 2 diabetics, together with reduced levels of specific antioxidants such as ascorbic acid and vitamin E [127]. Lipoic acid is an antioxidant capable of thiol-disulfide exchange. It is able to scavenge ROS and reduce metabolites such as glutathione to maintain a healthy cellular redox state [128]. This antioxidant attenuates the apoptosis of rat retinal capillary cells and decreases the levels of 8-OHdG and nitrotyrosine. Lipoic acid supplementation completely prevents diabetes-induced increase in nitrotyrosine and activation of NF-*κ*B while decreasing the levels of VEGF and oxidatively modified proteins in the rat retina [129]. Apart from lipoic acids other experiments have also been tried in animal models, such as vitamin C and vitamin E. All of them have shown improved biological and pathological changes and prevented or slowed the progression of diabetic complications [130]. The potential benefit of vitamin E has been shown in DR by its free radical scavenger activity outside the cell through nonenzymatic mechanisms [100]. Trolox is a water soluble analog of vitamin E with potent antioxidant properties. Trolox is shown to partially prevent the loss of pericytes in diabetic rats via reducing membrane lipid peroxidation [131]. Another antioxidant, calcium dobesilate, decreased retinal permeability, stabilized BRB, and reduced overexpression of VEGF in diabetic rats [132]. Carotenoids are some of the powerful antioxidants, and diabetes decreases lutein and zeaxanthin levels in the serum and retina. Kowluru et al. investigated the effect of carotenoid containing nutritional supplements which prevented diabetic retinopathy and also maintained normal retinal function, mitochondrial homeostasis, and inflammatory mediators [133].

In bovine endothelial cells, hyperglycemia induced a significant increase in the hexosamine pathway which was blocked by an inhibitor of electron transport, a mitochondrial uncoupling agent (CCCP), and the expression of either UCP1 or SOD2 [126]. A second new class of mechanism-based potential therapeutic agents is PARP inhibitors. In cultured arterial endothelial cells, a specific PARP inhibitor prevents hyperglycemia-induced activation of PKC, NF-*κ*B, intracellular AGE formation, and the hexosamine pathway. In animal models of diabetes, PARP inhibition prevents arterial endothelial cell injury and podocyte apoptosis, ameliorates nephropathy, and alleviates sensory neuropathy [60].

### 4.5. Cannabidiol as an Emerging and Novel Therapeutic Modality

Diabetic retinopathy is characterized by the breakdown and neurotoxicity of blood-retinal barrier (BRB) which have been associated with oxidative stress and proinflammatory cytokines that may operate by activating their downstream target p38 MAP kinase. Cannabinoids are known to possess therapeutic properties including NMDA receptor-activation [134], inhibition of oxidation [135], and inflammation (Buckley NE). (−)-Δ9-tetrahydrocannabinol (THC) and (−)-cannabidiol (CBD) are the marijuana-derived cannabinoids which possess antioxidative and immunosuppressive effects [136]. It has already been established that nonpsychotropic CBD causes a decrease in interleukin-1, TNF-*α*, and interferon-*γ* in murine collagen-induced arthritis and prevents central nervous system neuronal damage in gerbils [137]. El-Remessy et al. demonstrated the neuroprotective role of both THC and CBD through antioxidant action in NMDA-induced retinal neurotoxicity in rats [138]. They also demonstrated the BRB-preserving effects of blocking oxidative stress in diabetic rats. These studies emphasize the need for rational and conceptual considerations on the mode of action of CBD in the treatment of diabetic retinopathy, which can serve as a potential biomarker and novel therapeutic agent for diabetic retinopathy.

### 4.6. Role of the Renin-Angiotensin System, Its Inhibitors, and Use of Fenofibrate in Diabetic Retinopathy

The renin-angiotensin system (RAS) has an important role in regulation of electrolyte balance, vasoconstriction, and vascular remodeling. Local renin-angiotensin regulation is present in the eye [139]. The role of the RAS in diabetic retinopathy has not been as well studied as that in the kidney and nephropathy; however, several studies including the Renin-Angiotensin System Study (RASS), Daily-Dose Consensus Interferon and Ribavirin: Efficacy of Combined Therapy (DIRECT) trial, and the ADVANCE Retinal Measurements (AdRem) have provided evidence that RAS inhibition may also be beneficial in diabetic retinopathy [140, 141]. Studies have reported that ACE inhibitor improves endothelial function and stimulates vascular remodeling, in addition to attenuating progression of arteriosclerosis and the occurrence of cardiovascular events in humans [142, 143]. EURODIAB Controlled Trial of Lisinopril in Insulin-Dependent Diabetes Mellitus (EUCLID) study group reported a reduction in proliferative diabetic retinopathy via ACE inhibition, providing a potential clinical role for suppression of the renin-angiotensin system (RAS) in preventing and treating retinal neovascularization [144]. Satofuka et al. investigated receptor-associated prorenin system (RAPS) which dually activates the tissue renin-angiotensin system (RAS) and RAS-independent intracellular signaling. Their results showed a significant contribution of the RAPS to the pathogenesis of diabetes-induced retinal inflammation, suggesting the possibility of the (pro) renin receptor as a novel molecular target for the treatment of diabetic retinopathy [145]. From these studies we conclude that RAS blockade has an additional significant impact on slowing or stopping diabetic nephropathy and a more modest but still clinically relevant impact on diabetic retinopathy.

Fenofibrate is a peroxisome proliferactor-activated receptor- (PPAR-) *α* agonist that is used to treat high triglycerides and low HDL or as adjunct to statin therapy. It regulates the expression of many genes that work against lipids, inflammation, angiogenesis, and cell apoptosis. Diabetic patients not only need to maximize glycemic control, but also to closely monitor and treat other systemic conditions including diabetic retinopathy. Studies have shown consistent beneficial effects with fenofibrate in slowing the progression of DR [146]. Fenofibrate treatment among patients with type 2 diabetes reduced the need for laser treatment for diabetic eye disease. The mechanism by which this happens remains unclear; however, it does not appear to be related to plasma lipid concentrations. The ACCORD Eye Study group involved a subset of 2,856 participants and analyzed the effects of the treatment strategies on blood vessels in the eye by identifying diabetic retinopathy progression over four years. According to their report, rates of progression of diabetic retinopathy were significantly reduced in the intensive glycaemic control group and in the fenofibrate group, but not in the intensive blood pressure control group [147]. Keech et al. in FIELD (Fenofibrate Intervention and Event Lowering in Diabetes) study have reported that fenofibrate could reduce the need for laser treatment in a large cohort of 9,795 type 2 diabetic patients. Fenofibrate reduced the frequency of laser treatment for macular edema by 31% and for proliferative retinopathy by 30%. In addition, in a substudy performed on patients in whom retinal status was graded by fundus photography, fenofibrate was able to reduce the progression of existing retinopathy [148]. Although this study has some limiting factors, the substantial benefits obtained from reducing the need for laser treatment argue for consideration of using fenofibrate in the management of diabetic retinopathy [149]. The FIELD findings are clearly important therapeutically, but the trial's lasting contribution might be to provoke further research into underlying mechanisms of action of fenofibrate to improve endothelial function and reducing local inflammatory processes which could lead to new treatments of diabetic retinopathy.

## 5. Conclusion

The intent of this review is to provide better understanding of the complex molecular mechanisms and treatment modalities. At primary stage patients should be encouraged to keep a close eye on their blood pressure, duration of the disease, hyperlipidemia, metabolic control, and hypertension. To reduce and restore the vision loss, scatter laser photocoagulation or vitrectomy surgery can be employed. At the proliferative stages of the disease, the therapeutic interventions are effective in reducing visual loss; however, once DR develops, additional mechanisms, including hypoxia-induced VEGF production, contribute to retinal disease progression. Vascular endothelial growth factor is the most well-studied component of the mechanisms involved in the diabetic retinopathy and anti-VEGF therapies. Systemic therapeutic interventions targeting VEGF would be intravitreally administered so as to avoid the development of impaired angiogenesis in other organs. Hyperglycaemia induced de novo synthesis of DAG in vascular cells leads to selective activation of PKC isozymes, especially PKC-b, which in turn phosphorylate proteins involved in endothelial function and neovascularisation. These changes activate intracellular signaling proteins such as PKC, PKB, AGE, and MAPK which are finally culminating in pathological induction of transcription factors such as NF-*κ*B and AP-1. After reviewing these as well as data in the literature, it is evident that each of the four main mechanisms implicated in the pathogenesis of diabetic complications reflects a single hyperglycaemia-induced process and pathogenetic mechanisms involved in diabetic retinopathy are interactive and interdependent. Therefore, within the near future pharmacologic treatment strategies may include multiple pharmacological agents, targeted simultaneously to block multiple pathways. This will probably be available for treating and preventing the progression of DR after further potential clinical trials.

## Figures and Tables

**Table 1:** Diabetic retinopathy: clinical trials [9].

Number	Title	Target sample size	Study type	Country
1	Continuous Positive Airway Pressure (CPAP) in Patients with Impaired Vision due to Diabetic Retinopathy and Concurrent Obstructive Sleep Apnoea (OSA): ROSA trial	150	Interventional	UK

2	Screening intervals for diabetic retinopathy	24,000	Observational	UK

3	Computer Detection of Diabetic Retinopathy Compared to Clinical Examination	600	Observational	USA

4	Computer-based Screening for Diabetic Retinopathy	10,000	Observational	USA

5	Prompt Panretinal Photocoagulation Versus Ranibizumab + Deferred Panretinal Photocoagulation for Proliferative Diabetic Retinopathy	316	Interventional	USA

6	Treatment for CI-DME in Eyes With Very Good VA Stud	702	Interventional	USA

7	The Role of Prostaglandins in the Progression of Diabetic Retinopathy	100	Interventional	USA

8	Comparison of Phase-variance Optical Coherence Tomography and Fluorescein Angiography in Retinovascular Imaging	78	Observational	USA

9	The Use of Alpha Lipoic Acid for the Treatment and Prevention of Diabetic Retinopathy	200	Interventional	USA

10	NSAID Phase II for Non-central Involved Diabetic Macular Edema (DME)	120	Interventional	USA

11	Rapid, Non-invasive, Regional Functional Imaging of the Retina. (Diabetic Retinopathy Diagnosis Device)	315	Observational	USA

12	A Safety and Efficacy Study of Vitreosolve for Non-Proliferative Diabetic Retinopathy Subjects	160	Interventional	India

13	Intravitreal Bevacizumab for Retinal Disorders	150	Interventional	India

14	A study to Find out Whether Vitrectomy Is Better Than Laser for Diabetic Patients with Partial Bleeding into the Vitreous Jelly	64	Interventional	India

15	Anterior and Posterior Segment Vascular Changes Following Laser and Anti-Vascular Endothelial Growth Factor (VEGF) Treatment of Diabetic Retinopathy	64	Interventional	Canada

16	Prospective Study Phase: Retinal Oxygen Saturation, Blood Flow, Vascular Function and High Resolution Morphometric Imaging in the Living Human Eye	381	Observational	Canada

17	A Comparison of Islet Cell Transplantation With Medical Therapy for the Treatment of Diabetic Eye Disease	40	Interventional	Canada

18	Prospective Study Phase: Retinal Oxygen Saturation, Blood Flow, Vascular Function and High Resolution Morphometric Imaging in the Living Human Eye	381	Observational	Canada

19	Safety and Efficacy of Low-Fluence PRP for PDR	60	Interventional	Mexico

20	Topic Antiinflammatory Therapy Added to Selective Photocoagulation in Macular Edema	84	Interventional	Mexico

21	Standard versus Intensive Statin Therapy for Hypercholesterolemic Patients with Diabetic Retinopathy	5,000	Interventional	Japan

22	Japan Public Health Center-based Prospective Study-NEXT on Glaucoma, Age-Related Macular Degeneration and Diabetic Retinopathy	4,000	Observational	Japan

23	Research on Age-Related Macular Degeneration and Diabetic Complications Using Non-invasive AGEs Measurement Equipment	500	Observational	Japan

24	Choroidal Structure of Diabetic Retinopathy Eye on OCT Image After the Treatment	50	Interventional	Japan

25	Diabetes-Related Eye Disease Study	400	Observational	Japan

26	The Effect of NSAID for the Cystoid Macular Edema After Panretinal Photocoagulation in Diabetic Retinopathy	140	Interventional	Japan

27	To Investigate the Effects of Panretinal Photocoagulation by a Multicolor laser Photocoagulator with a Scan Delivery System in Eyes with Proliferative Diabetic Retinopathy	60	Interventional	Japan

28	A Pilot Study on the Effects of ILARIS on Patients With Proliferative Diabetic Retinopathy (PDRP)	10	Interventional	Switzerland

29	Panretinal Photocoagulation for Diabetic Retinopathy With PASCAL Laser	60	Interventional	Brazil

30	Bevacizumab as Adjunctive Treatment to Laser Panretinal Photocoagulation for Proliferative Diabetic Retinopathy	30	Interventional	Brazil

31	Thiazolidinedione (TZD) on the Diabetic Retinopathy and Nephropathy	200	Interventional	Taiwan

32	Genetic Association of Diabetic Retinopathy-1	200	Observational	Taiwan

33	Retinal Adaptation to Intensified Insulin Therapy and Bariatric Surgery in Patients With Diabetes	100	Observational	Denmark

34	Topical Application of Latanoprost in Diabetic Retinopathy	50	Interventional	Denmark

35	A Phase 2 Clinical Study to Investigate Effects of Darapladib in Subjects With Diabetic Macular Edema	54	Interventional	Australia

36	Trial of Switching Between Intravitreal Bevacizumab (Avastin) & Intravitreal Dexamethasone (Ozurdex) for Persistent Diabetic Macular Oedema	50	Interventional	Australia

37	Intravitreal Triamcinolone Acetonide for Diabetic Macular Edema	60	Interventional	Germany

38	Multicenter 12 Months Clinical Study to Evaluate Efficacy and Safety of Ranibizumab Alone or in Combination With Laser Photocoagulation vs. Laser Photocoagulation Alone in Proliferative Diabetic Retinopathy (PRIDE)	120	Interventional	Germany

39	Effects of Fenofibrate on Endothelial Progenitor Cells in Type 1 Diabetes	38	Interventional	Italy

40	Effect of Folic Acid, Vitamin B6 and Vitamin B12 in Diabetic Retinopathy	160	Interventional	Italy

41	Intravitreal Ozurdex After Pars Plana Vitrectomy for Proliferative Diabetic Retinopathy	100	Interventional	Sweden

42	Vitreous Analysis in Proliferative Diabetic Retinopathy	200	Observational	Sweden

43	Different Interventions Promoting Diabetic Retinopathy Screening Among Chinese Type 2 diabetes: A Randomized Trial	300	Interventional	China

44	Clinical Investigation on Early Lesions in Diabetic Retinopathy	500	Observational	China

45	Laser Photocoagulation in Patients with Diabetic Retinopathy Derived from New International Clinical Classification	180	Observational	China

46	Effect of Berberine on Diabetic Retinopathy	100	Interventional	China

47	Study of Evaluation on the Clinical Efficacy of Tradition Chinese Medicine in the Treatment of Non-Proliferative Diabetic Retinopathy	60	Interventional	China

48	Clinical Study of Treatment with Kudiezi Injection in Nonproliferative Diabetic Retinopathy Patients	80	Interventional	China

49	Hemodynamics of Ocular Artery in Ischemic Ocular Diseases with TCD Study	180	Diagnostic test	China

50	Morphological and Functional Retinal Changes Following Retinal Photocoagulation	50	Interventional	Austria

51	Study Investigating the Levels and Effects of Low-grade Inflammation in Diabetic Retinopathy of Type 1 Diabetes	50	Observational	Austria

52	Choroidal Blood Flow Changes During Dark/Light Transitions in Patients With Insulin-Dependent Diabetes Mellitus (IDDM)	80	Interventional	Austria

53	Autologous Plasmin and Fibrinolytic System in Diabetic Retinopathy	40	Interventional	Korea, Republic of Korea

54	Incidence of Macular Edema After Panretinal Photocoagulation (PRPC) Performed in a Single Session Versus Four Sessions in Diabetic Patients.	90	Interventional	France

55	Trial of Yellow 577 nm Laser Versus Green 532 nm Laser for Proliferative Diabetic Retinopathy	120	Interventional	Malaysia

56	Preoperative Injection of Bevacizumab Prior to Vitreoretinal Surgery in Diabetic Tractional Retinal Detachment	50	Interventional	Iran, Republic of Iran

57	Effect of Intravitreal Bevacizumab on Early Post-Vitrectomy Hemorrhage in Diabetic Patients	80	Interventional	Iran, Republic of Iran

58	Intravitreal Adalimumab in Refractory Diabetic Retinopathy, Choroidal Neovascularization or Uveitis: A Pilot Study	15	Interventional	Lebanon
